# Deep Neural Network to Accurately Predict Left Ventricular Systolic Function Under Mechanical Assistance

**DOI:** 10.3389/fcvm.2021.752088

**Published:** 2021-10-26

**Authors:** Jean Bonnemain, Matthias Zeller, Luca Pegolotti, Simone Deparis, Lucas Liaudet

**Affiliations:** ^1^Department of Adult Intensive Care Medicine, Lausanne University Hospital and University of Lausanne, Lausanne, Switzerland; ^2^SCI-SB-SD, Institute of Mathematics, School of Basic Sciences, Ecole Polytechnique Fédérale de Lausanne, Ecublens, Switzerland

**Keywords:** cardiovascular modeling, heart failure, deep neural network, left ventricular assist device, machine learning

## Abstract

Characterizing left ventricle (LV) systolic function in the presence of an LV assist device (LVAD) is extremely challenging. We developed a framework comprising a deep neural network (DNN) and a 0D model of the cardiovascular system to predict parameters of LV systolic function. DNN input data were systemic and pulmonary arterial pressure signals, and rotation speeds of the device. Output data were parameters of LV systolic function, including end-systolic maximal elastance (E_*max,lv*_), a variable essential for adequate hemodynamic assessment of the LV. A 0D model of the cardiovascular system, including a wide range of LVAD settings and incorporating the whole spectrum of heart failure, was used to generate data for the training procedure of the DNN. The DNN predicted E_*max,lv*_ with a mean relative error of 10.1%, and all other parameters of LV function with a mean relative error of <13%. The framework was then able to retrieve a number of LV physiological variables (i.e., pressures, volumes, and ejection fraction) with a mean relative error of <5%. Our method provides an innovative tool to assess LV hemodynamics under device assistance, which could be helpful for a better understanding of LV-LVAD interactions, and for therapeutic optimization.

## 1. Introduction

Left Ventricular Assist Device (LVAD), a subset of mechanical circulatory support, assists the failing left ventricle (LV) by pumping blood from the LV into the ascending aorta. In recent years, LVAD has become a crucial therapeutic solution for patients with end-stage heart failure (1). Current indications for LVAD implantation include bridge to heart transplantation (2), and destination therapy, for patients not candidate for heart transplantation (3). LVAD may also be used as bridge to recovery, in patients in whom the LVAD can be removed after recovery of native myocardial function (4).

The assisted LV may retain a certain amount of residual native function. Therefore, complex and reciprocal interactions occur between the heart and the LVAD. The systemic blood flow, i.e., total cardiac output, represents the sum of the flows generated by the LVAD and the native LV, dependent on respective loading conditions (preload and afterload) and inherent function. The latter corresponds to the rotational speed for the device (revolutions per minute, RPM) and residual systolic function (contractility or inotropy) of the native LV (5). Understanding these interactions is critical for appropriate settings of the LVAD and for estimating the possible recovery of native LV function. An essential step is therefore to appropriately evaluate LV systolic function under LVAD assistance (6).

Such evaluation is particularly challenging, owing to the unloading of the LV produced by the LVAD. Echocardiography and invasive cardiac catheterization are presently the only methods used for this purpose, and some authors have advocated specific protocols to optimize native LV function or to identify myocardial recovery (7–10) in patients assisted by LVAD. These methods, while clinically useful, provide either indirect (catheterization) or load-dependent indices of LV function, but unfortunately do not provide a direct, load-independent determination of LV contractility. The latter can indeed only be fully characterized by computing maximal systolic elastance (*E*_*max,lv*_), which is the maximal slope of the end-systolic pressure volume relationship of the LV (11). Determining *E*_*max,lv*_ requires the simultaneous measurement of LV pressure and LV volume. This is only feasible under experimental settings and is therefore not applicable to the clinical reality, indicating that novel strategies to assess LV inotropy are critically needed.

We recently proposed a method, based on a deep neural network (DNN), to predict *E*_*max,lv*_ in failing, unassisted LV (12). In the present study, we aimed at evaluating whether such an approach could be used in the setting of LVAD support. To address this issue, we developed a framework using simple physiological signals (arterial pressure waveforms from the systemic and pulmonary arterial circulations) coupled to LVAD data (pump rotational speed ω_*c*_) to predict *E*_*max,lv*_ and other variables relevant to LV systolic function. The presence of the LVAD strongly affects the arterial pressure waveforms, it is therefore not possible to employ the same DNN considered in Bonnemain et al. (12) to recover the physiological variables of interest. In other words, the inherently different behavior of our cardiovascular system model (a 0D lumped model, see section 2.2) with and without LVAD requires us to train a new DNN on data specifically accounting for the presence of the device. One of the goals of this work, therefore, is to demonstrate that the approach presented in Bonnemain et al. (12) can be extended to patients with LVAD support by training the DNN on data adequately representing the pump settings considered in clinical settings.

## 2. Methods

### 2.1. General Framework

**Figure 1:**
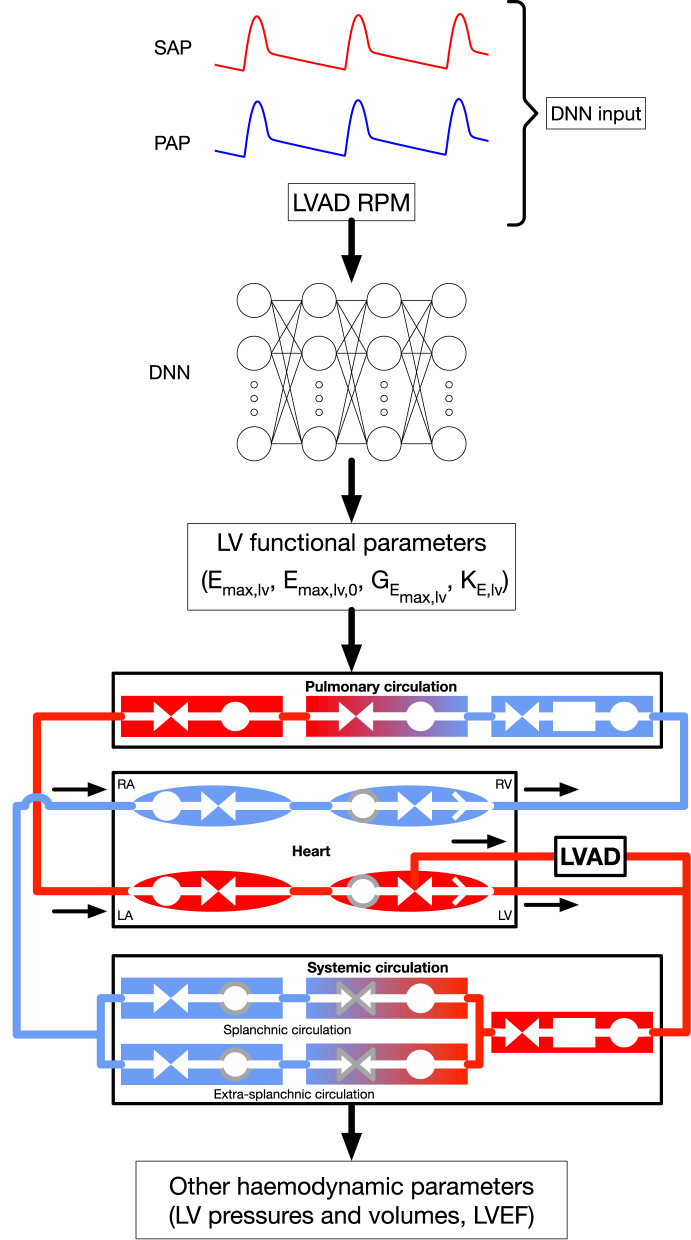
General framework, modified from Bonnemain et al. (12) under CC-BY license. The DNN is fed with systemic and pulmonary arterial pressures, formulated in their frequency domain, as well as LVAD RPM, to predict parameters of LV systolic function. These parameters are integrated to the 0D model to retrieve the indicated additional hemodynamic parameters. Shapes and colors significance in the 0D model are: deoxygenated blood (blue elements); oxygenated blood (red elements:); compliance (circles); inertance (rectangles); resistance (facing triangles, double arrows); cardiac valves (single white arrows in heart chambers); elements affected by autoregulation (gray contour). RPM, Rotations per minute; SAP, systemic arterial pressure; PAP, pulmonary arterial pressure; DNN, deep neural network; RA, right atrium; RV, right ventricle; LA, left atrium; LV, left ventricle. *E*_*max,lv*_ [mmHg/ml]: end-systolic left ventricular elastance, *E*_*max,lv*,0_ [mmHg/ml]: end-systolic left ventricular elastance in absence of baroregulation, *G*_*E*_*max,lv*__ [mmHg/ml/(spikes/ml)]: maximum baroreceptor gain, *k*_*E,lv*_ [1/ml]: steepness of end-diastolic pressure-volume curve.

Figure 1 displays a schematic representation of the framework (based on our recent publication (12)), used to evaluate (i) LV parameters of systolic function (*E*_*max,lv*_ and other parameters of LV systolic function) and (ii) various physiological LV quantities (e.g., pressures, volumes), by employing a lumped parameter model of the cardiovascular system (see description below). Our framework makes use of a deep neural network (DNN) which infers, given systemic and pulmonary arterial pressure measurements and the RPM of the LVAD as inputs, an estimation of LV systolic parameters (comprising, in particular, *E*_*max,lv*_). The DNN was trained with a lumped model of the cardiovascular system (13), modified to take into account the presence of a HeartMate III (HMIII) LVAD (Abbott Laboratories), one of the last third generation centrifugal-flow devices. In a second phase, the parameters of LV systolic function—which in general are unavailable in the daily practice—were provided to the lumped model to reconstruct various outputs such as LV pressures and volumes. It is worth noting that the network was trained on a dataset which is generated by the 0D model for a variety of heart failure cases and LVAD settings, therefore the first phase of the algorithm effectively consists in solving an inverse problem mapping the output of the 0D model to its underlying physical parameters.

### 2.2. Lumped Model of the Cardiovascular System and LVAD Modelization

The 0D model of the cardiovascular system is based on the mathematical description of the cardiovascular system presented by Ursino (13), which takes into account carotid baroregulation. It includes vascular compartments, heart ventricles with time-varying elastance, parasympathetic afferent and efferent pathways, and sympathetic efferent pathway. We modified it in order to model the presence of the HMIII LVAD.

The model comprises eight vascular compartments. The pulmonary circulation is represented by a serial arrangement of arterial, peripheral, and venous circulations. The systemic circulation begins with arteries and further subdivides into splanchnic and extrasplanchnic circulations, each having a peripheral and venous compartment in series. Each compartment *i* comprises at least a resistance (*R*_*i*_) and a compliance (*C*_*i*_) to account for viscous and elastic effects, respectively, and is further characterized by its unstressed volumes. Inertance (*L*_*i*_) is considered only in large systemic and pulmonary arteries, i.e., blood acceleration is neglected in the rest of the vascular tree. Mass conservation (1), momentum conservation (2), and pressure-flow relationship (3) equations are applied to each compartment *i*, assuming that blood is an incompressible, isotropic, and Newtonian fluid:


(1) $$\begin{array}{l} \frac{dV_{i}}{dt}\left(t\right)=Q_{i,in}\left(t\right)-Q_{i,out}\left(t\right), \end{array}$$

(2) $$\begin{array}{l} L_{i}\frac{dQ_{i,out}}{dt}\left(t\right)=P_{in}\left(t\right)-P_{out}\left(t\right)-R_{i}\left(t\right)Q_{i,out}\left(t\right), \end{array}$$

(3) $$\begin{array}{l} V_{i}\left(t\right)=C_{i}P_{i,in}\left(t\right)+V_{i,0}, \end{array}$$

where *V*_*i*_ is the volume of *i*th compartment, *V*_*i*,0_ the unstressed volume of *i*th compartment (i.e., volume at zero pressure), *Q*_*i,in*_ and *Q*_*i,out*_ the inlet and outlet flow rates of *i*th compartment, *P*_*i,in*_ and *P*_*i,out*_ the inlet and outlet pressures of *i*th compartment.

The heart model comprises ventricles and atria as a four-compartment system, each of which is modeled by a serial arrangement of a compliance, a resistance, and an ideal valve, i.e., blood flows without viscous loss from inlet to outlet compartments when pressure of the former exceeds pressure of the latter. Atria are passive elements, whereas contractility of ventricles is characterized by a time-varying elastance. Autoregulation occurs through the carotid baroreflex. The vagal afferent activity is modulated by the absolute systemic arterial pressure and its rate of change. Sympathetic and vagal efferent activities then modulate systemic peripheral resistances, systemic venous compliances, heart period, and ventricles resistances and compliances.

**Figure 2:**
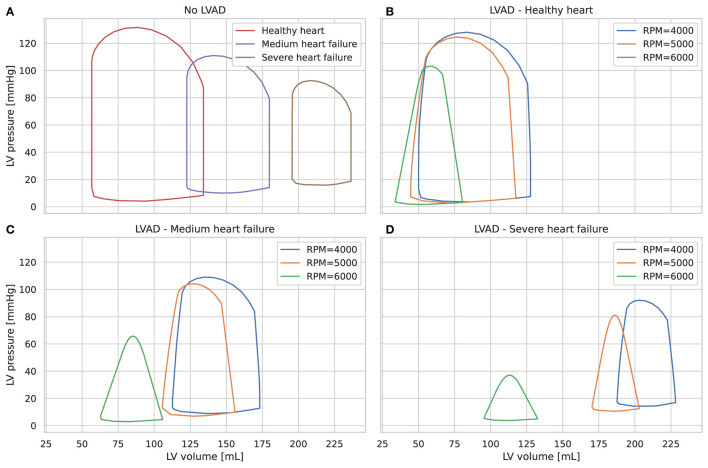
Left ventricle pressure-volume diagrams for different stages of heart failure, without LVAD **(A)** and with LVAD for different pump speeds **(B–D)**.

Different stages of left ventricle systolic failure severity were represented by modifying values of the following parameters: end-systolic elastance of the left ventricle with and without autoregulation, *E*_*max,lv*_ and *E*_*max,lv*,0_, respectively, the maximum baroreceptor gain *G*_*E*_*max,lv*__, and *k*_*E,lv*_, which describes the end-diastolic pressure-volume relationship for the left ventricle. Ranges for each parameters are shown in Supplementary Table 1 and were validated with clinical data in Bonnemain et al. (14). All other parameters were not changed and can be found in the original paper (13). Figure 2 shows different pressure-volume diagrams for different degrees of heart failure, with and without LVAD.

The LVAD is modeled as a pressure-controlled flow generator, based on pressure-flow curves interpolated from data available in the HMIII manual^1^. Specifically, the flow rate is a function of the pump differential pressure and the pump rotational speed, as shown in Supplementary Figure 1. The LVAD inflow and outflow cannulas are connected to the left ventricle and systemic arteries, respectively, as depicted at the bottom of Figure 1. The pump setting is characterized by a constant rotational speed, ω_*c*_, and a pump speed modulation feature, namely the artificial pulse, which periodically modifies the pump speed from its preset value ω_*c*_. More specifically, every 2 s the pump speed decreases by 2,000 RPM during 0.15 s and then increases by 4,000 RPM during the following 0.2 s. This aims at promoting pump washout (15) and thus preventing pump thrombosis (3, 5).

### 2.3. Deep Neural Networks

A DNN is a parametric machine learning algorithm used to capture complex nonlinear relationships between inputs and outputs, which needs to be trained on a large number data points. In contrast to other parametric models, DNNs do not require strong assumptions about the nature of the data distribution. The fundamental building block of a DNN is an artificial neuron, that is a simple function which takes a *d*-dimensional input **x** = (*x*_1_, …, *x*_*d*_) and which outputs a scalar *a* = *g*(*w*_0_ + *w*_1_*x*_1_ + *w*_2_*x*_2_ + … + *w*_*d*_*x*_*d*_), where the vector $\text{w}=\left(w_{0},\ldots ,w_{d}\right)\in {\mathbb{R}}^{d+1}$ contains the parameters of the model, and *g* is the activation function, which is typically non-linear. Here, we focus on a simple and widely-used class of DNNs, namely multi-layer perceptrons (MLPs). A MLP is made of layers composed of artificial neurons in which each neuron receives input from all neurons in the previous layer; for this reason, this structure is commonly referred to as fully-connected. In matrix form, the output of the *l*th layer reads


(4) $$\begin{array}{l} {\text{a}}^{\left(l\right)}={\text{g}}^{\left(l\right)}\left(W^{\left(l\right)}{\text{a}}^{\left(l-1\right)}+{\text{b}}^{\left(l\right)}\right), \end{array}$$

where *W*^(*l*)^ and **b**^(*l*)^ are the weight matrix (which collects all the parameters **w** of the neurons belonging to the layer) and the bias vector, respectively. We denote by *L* the total number of layers of the DNN. Layer 0 is the DNN input, and layer *L* is the output, whereas layers 0 < *l* < *L* are called hidden layers.

Each layer *l* in a MLP depends on the parameters contained in the weight matrix *W*^(*l*)^ and in the bias vector **b**^(*l*)^. The process of calibration of these parameters corresponds to the training of the DNN. It is based on the use of a training dataset represented by a set of input-output pairs {**x**^*i*^, **y**^*i*^}. Let **f**(**x**^*i*^; Θ) = **a**^(*L*)^ when **a**^(0)^ = **x**^*i*^, where Θ = {*W*^(1)^, …, *W*^(*L*)^, **b**^(1)^, …, **b**^(*L*)^} is the set of parameters of all layers. The fitting or training procedure consists in minimizing a loss function $L_{\Theta }$ on the training set by means of an optimization algorithm (e.g., stochastic gradient descent). The goal is to find the weights Θ that yield good approximations **f**(**x**^*i*^; Θ) ≈ **y**^*i*^. See (16, 17) for a detailed description of machine learning algorithms and deep neural networks.

### 2.4. Data Generation and Input/Output of the DNN

We generated a dataset by solving the 0D model *N*_*s*_ times for *N*_ω_ pump rotational speeds ω_*c*_. Each of the *N*_*s*_ × *N*_ω_ simulations was characterized by a pump setting (ω_*c*_) and a set of four heart-failure-characterizing parameters (Supplementary Table 1), independently and randomly sampled with a uniform distribution. We selected *N*_ω_ = 21 pump rotational speeds ω_*c*_ equally spaced in the range from 4,000 to 6,000 RPM, as these speeds encompass the usual pump setting in clinical conditions (18). For each rotational speed ω_*c*_, *N*_*s*_ = 10, 000 samples were generated, yielding a total of *N*_ω_ × *N*_*s*_ = 210, 000 samples. Setting a large value for *N*_*s*_ allowed us to generate combinations of parameters that cover the whole range of left heart failure stages. The other 0D parameters are set to the values found in Ursino (13).

A simulation was performed over a time range (0, *T*) with *N* interpolation equidistant points. We set the simulation time to *T* = 30 s, allowing signals to reach a steady state after initialization, with *N* = 2, 000. The resulting output was a set of evenly-spaced time series. We note $u^{i,\text{SAP}}\left(t_{m}\right)$ and $u^{i,\text{PAP}}\left(t_{m}\right)$ the values of the systemic arterial pressure (SAP) and pulmonary arterial pressure (PAP), respectively, at time *t*_*m*_ = *mT*/*N, m* ∈ {0, 1, …,*N*}, for the *i*th sample of the dataset. The pressure curves were represented in the frequency domain. That is, we computed the trigonometric Fourier coefficients $a_{k}^{i,X}$ and $b_{k}^{i,X}$ such that,


$$u^{i,X}\left(t_{m}\right)=\frac{a_{0}^{i,X}}{2}+\overset{N/2}{\underset{k=1}{\sum }}\left[a_{k}^{i,X}\cos\left(\omega_{k}t_{m}\right)+b_{k}^{i,X}\sin\left(\omega_{k}t_{m}\right)\right],$$

where *N* is even, ω_*k*_ = 2*kπ*/*T* and *X* is either SAP or PAP (19).

We fixed the activation functions of the DNN and trained a model for various number of layers and neurons per layer. In addition to the Fourier coefficients of the signals, we provided the pump setting $\omega_{c}^{i}$ of *i*th sample as an additional predictor variable. To reduce the noise and to obtain better trainability thanks to a smaller input size, we choose a small number *K* and we only keep (2*K*−1) Fourier coefficients of the signal *u*^*i,X*^, which are stored in a vector ${\text{c}}^{i,X}=\left(a_{0}^{i,X},\ldots ,a_{K-1}^{i,X},b_{1}^{i,X},\ldots ,b_{K-1}^{i,X}\right)$. An input-output pair is represented by the input vector ${\text{x}}^{i}=\left({\text{c}}^{i,\text{SAP}},{\text{c}}^{i,\text{PAP}},\omega_{c}^{i}\right)$, and the output vector ${\text{y}}^{i}=\left(E_{max,lv}^{i},E_{max,lv,0}^{i},G_{E_{max,lv}}^{i},k_{E,lv}^{i}\right)$.

### 2.5. Software Implementation

The 0D model has been implemented in the object-oriented and equation-based Modelica programming language, on the open-source OpenModelica framework. Pre- and post-processing procedures were implemented in the Python and Matlab languages. The DNN architecture was implemented with the Keras library within TensorFlow.

## 3. Results

### 3.1. Fourier Coefficient Determination

We first aimed at determining the numbers of Fourier coefficients *K* required to accurately reconstruct the pressure signals. These are in turn given as input to the DNN. We provide in Supplementary Figure 2 a reconstruction of a pressure signal with different values of *K* for a complex curve, as obtained with low RPM (4,000) and severe LV failure. Indeed, in these conditions, LV preload is little reduced, allowing the native LV to contract and eject through the aortic valve. Thus, ejection through the LVAD and the native aorta induced a complex signal (20). In addition, artificial pulse of the device adds a level of perturbation, that is moreover not synchronized with heart rate. Black curve on the top of the figure corresponds to the original signal, and colored curves reconstructions with different values of *K*. We chose to restrict to *K* = 50. While limiting the size of the input data, this choice enables an accurate reconstruction of pressure curve signals.

### 3.2. DNN Architecture Evaluation

We used the rectified linear unit and sigmoid activation functions for the hidden and output layers, respectively. That is, *g*^(*l*)^(*x*) = max(*x*, 0) for *l* < *L* and *g*^(*L*)^(*x*) = *e*^*x*^/(1 + *e*^*x*^). For training, we used the typical mean squared error loss function $L_{\Theta }=\frac{1}{\left|B\right|}\underset{i\in B}{\sum }{\left[{\text{y}}^{i}-\text{f}\left({\text{x}}^{i};\Theta \right)\right]}^{2}$ that we minimized with Adam optimizer, with *B* = 32 being the batch size. Learning rate was set to 0.001. 5% of the samples in the dataset were kept for the test set, while 80% and 20% of the remaining samples were used in the training and validation sets, respectively.

We evaluated 28 different DNN architectures. For each architecture, the training and validation values of the loss and the mean absolute error were computed, as shown in Supplementary Table 2. We then selected the best performing and smallest architecture (in terms of number of layers and neurons), corresponding to architecture #2 in Supplementary Table 2.

### 3.3. DNN Performances

**Figure 3:**
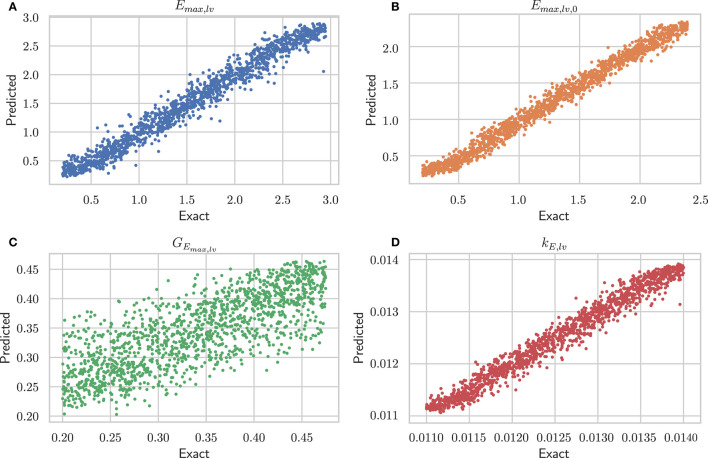
Performance of architecture #2 (see Supplementary Table 2) on the test set. For each of the four parameters, the exact value is plotted against the predicted one. **(A)** *E*_*max,lv*_ [mmHg/ml], end-systolic left ventricular elastance; **(B)** *E*_*max,lv*,0_ [mmHg/ml], end-systolic left ventricular elastance in absence of baroregulation; **(C)** *G*_*E*_*max,lv*__ [mmHg/ml/(spikes/ml)], maximum baroreceptor gain; **(D)** *k*_*E,lv*_ [1/ml], slope of end-diastolic pressure-volume curve.

Table 1 shows performances of the DNN to predict the 4 output parameters of the test set (10, 500 samples). The data indicate accurate predictions for *E*_*max,lv*_, *E*_*max,lv*,0_, and *k*_*E,lv*_ (mean relative error of predictions, respectively, 10.07, 7.58, and 0.93%). The predictive accuracy was slightly lower for *G*_*E*_*max,lv*__ (mean relative error 12.43%), which is consistent with the sensitivity analysis performed in Bonnemain et al. (12), showing a lower sensitivity of the 0D model to this parameter. Graphical representation of data presented in Table 1 is provided in Figure 3.

**Table 1:** Evaluation of the DNN performance on the test set, by comparing exact and predicted parameters of the output of the DNN.

	***E*_*max,lv*_**	***E*_*max,lv*,0_**	***G*_*E*_*max,lv*__**	***k*_*E,lv*_**
**Exact**				
Min	0.200	0.200	0.200	0.0110
Max	2.95	2.39	0.475	0.0140
Mean	1.58	1.30	0.338	0.0125
SD	0.791	0.632	0.0794	8.65e-4
**Predicted**				
Min	0.204	0.208	0.203	0.0111
Max	2.91	2.38	0.472	0.0140
Mean	1.56	1.27	0.342	0.0125
SD	0.782	0.627	0.0632	8.42e-4
**Error**				
Mean	0.111	0.0693	0.0386	1.17e-4
Min	2.05e-5	1.24e-6	2.12e-5	6.85e-9
Max	0.913	0.352	0.202	9.88e-4
SD	0.0887	0.0524	0.0319	9.56e-5
CI min	0.109	0.0683	0.0380	1.15e-4
CI max	0.112	0.0703	0.0392	1.18e-4
**Relative error**				
Mean	0.101	0.0758	0.124	9.33e-3

*Error is computed as the difference between exact and predicted output in absolute value. Relative error is error divided by exact output. E_max,lv_ [mmHg/ml], end-systolic left ventricular elastance; E_max,lv,0_ [mmHg/ml], end-systolic left ventricular elastance in absence of baroregulation; G_E_max,lv__ [mmHg/ml/(spikes/ml)], maximum baroreceptor gain; k_E,lv_ [1/ml], steepness of end-diastolic pressure-volume curve*.

The DNN performance was further assessed by solving the 0D model with the 4 parameters (real and predicted) for each sample of the test set, using the following output measures: LV end-systolic pressure and volume, LV end-diastolic pressure and volume, LV ejection fraction, and pulmonary artery wedge pressure (a clinical surrogate of LV end-diastolic pressure). For each variable, the minimal, maximal, and mean values, as well as the standard deviation, were obtained using alternatively the exact and predicted values of the 4 parameters of LV systolic function, and the differences between obtained data were computed as the error, presented as mean ±SD, 95% confidence interval, and relative error. The results of this analysis are presented in Table 2 which indicates values of relative error <5% for all the hemodynamic values evaluated.

**Table 2:** Results of 0D simulations using exact and predicted parameters of the test set.

	**LVEF**	**LVEDV**	**LVESV**	**LVEDP**	**LVESP**	**PCWP**
**Exact**						
Mean	53.0	141	67.8	3.66	113	7.43
Min	24.4	86.0	28.7	1.03	39.0	3.11
Max	71.1	220	166	12.0	135	16.7
SD	9.16	22.0	22.5	1.70	14.1	2.30
**Predicted**						
Mean	52.6	142	68.9	3.71	112	7.49
Min	24.3	85.6	29.2	1.05	39.2	3.15
Max	70.1	217	159	12.9	135	17.3
SD	9.30	22.6	23.3	1.75	14.5	2.34
**Error**						
Mean	1.04	1.62	2.15	0.112	0.881	0.107
Min	1.17e-4	8.11e-5	5.83e-7	9.44e-7	5.53e-6	2.14e-5
Max	8.36	16.8	17.5	1.27	20.4	1.09
SD	1.04	1.52	2.15	0.127	1.39	0.109
CI min	1.02	1.59	2.11	0.109	0.855	0.105
CI max	1.06	1.65	2.19	0.114	0.908	0.109
**Relative error**						
Mean	0.0204	0.0111	0.0317	0.0295	8.82e-3	0.0138

*The error on the retrieved haemodynamic parameters is expressed as absolute or relative. LVEF [%], left ventricular ejection fraction; LVEDP [mmHg], left ventricular end-diastolic pressure; LVESP [ml], left ventricular end-systolic pressure; LVEDV [ml], left ventricular end-diastolic volume; LVESV [ml], left ventricular end-systolic volume; PCWP [mmHg], pulmonary capillary wedge pressure*.

## 4. Discussion

LVAD has become a frequently used therapeutic option in end-stage heart failure. Although this type of mechanical circulatory support significantly improves clinical condition and outcome of patients (21), some issues deserve further exploration regarding the interactions between the LVAD and the residual function of the native LV (22). A better understanding of these interactions and of hemodynamic properties of the assisted LV would be important to optimize support strategies, detect early abnormal interactions between device and LV, and finally identify LV recovery to consider weaning of the LVAD (23).

Our present work provides a novel approach to help address such complex issues by implementing a DNN and by using a 0D model of the cardiovascular system, which incorporates the mathematical description of a last generation LVAD. We developed an automated framework to accurately recover LV hemodynamic parameters from data available in the clinical practice, namely systemic and pulmonary arterial pressures. These signals were represented in their frequency domain and were then given as input to the DNN. An appropriate selection of the number of Fourier coefficient allowed to retain only relevant physiological frequencies, control size of input, and clean possible noise. In addition to the pressure signals, the input of the DNN included information about the device setting (RPM).

Owing to this architecture, only one DNN has to be trained to include every possibility of the working range of the device. This makes our framework suitable for a fast and automated implementation. Our DNN proved excellent reliability, being able to predict *E*_*max,lv*_ with a mean relative error of <10%. Furthermore, the 0D model allowed to precisely recover values of LVEF, ventricular volumes, and ventricular pressures, as indicated by the small relative error (<5%) between their actual and predicted values. Moreover, the average time to predict LV parameters from input signal was fast (<1 s) using a personal computer. Thus, real-time implementation could be easily considered.

The determination of *E*_*max,lv*_ is challenging, requiring left-side heart chambers catheterization, a technique whose implementation is not realistic in the daily clinical practice. Non-invasive methods, including echocardiography and magnetic resonance imaging, coupled to measurement of arterial blood pressure have been proposed (24, 25), however their use is mainly restricted to the experimental setting. Furthermore, none of these techniques have so far been applied to assess *E*_*max,lv*_ in patients assisted with a LVAD. It is noteworthy that a few preclinical studies have highlighted significant difficulties to determine *E*_*max,lv*_ in the presence of a LVAD. In a study performed using an *in vitro* cardiac simulator under control and heart failure conditions, Jhune et al. (26) showed that acute LVAD support induced a “pseudo-improvement” of calculated ventricular elastance, highly dependent on the LVAD speed. In two experimental animal studies, a comparable dependence of ventricular elastance on LVAD pump speed has also been reported by Vandenberghe et al. (27) in a calf model, whereas Sugai et al. (28) did not find such dependence in a goat model.

In the present work, the determination of *E*_*max,lv*_ remained accurate whatever the degree of residual LV function, LVAD setting, and loading conditions. Therefore, our method has the ability to determine *E*_*max,lv*_ independently from all potential influences of the aforementioned parameters, thereby avoiding misleading information. The framework was able to generate large amounts of data encompassing the whole working range of the device and every stage of heart failure severity, thereby permitting to appropriately train the DNN and guarantee the accuracy of predictions. Therefore, the implementation of our framework allowed to leverage the power of DNN to predict key parameters whose determination would be otherwise extremely cumbersome. An important issue to emphasize here is that the value of *E*_*max,lv*_ retrieved with our DNN cannot be calibrated with an effective measurement, which should require simultaneous recordings of intracardiac pressure and volume. Therefore, the absolute value of *E*_*max,lv*_ as obtained from our framework, should be interpreted with caution, whereas variations of this value along time would provide invaluable information to identify rapidly changing hemodynamic condition such as can occur in the acute setting.

The implementation of our tool could be notably useful in the post-operative phase of LVAD implantation. Indeed, in this context, LVAD RPM must be constantly adjusted to find the optimal settings of the device: although too high RPMs may lead to suction events promoting a reduction of pump flow, too low RPMs may cause inadequately low LVAD flow with systemic hypoperfusion, as well as left ventricle insufficient unloading and pulmonary oedema. These disturbances are likely to be influenced by the residual left ventricular systolic function, whose real time evaluation using our method would therefore be extremely helpful for hemodynamic optimization (29–31). Owing to the rapidity of the framework to make predictions on low-performance devices (e.g., standard personal computer), its direct implementation in the monitoring system of the patient might be straightforwardly considered. Arterial signals could be analyzed to make real-time predictions of LV parameters. Moreover, further development may include automatized algorithm to optimally set RPM in function of the predictions. This concept could be already implemented in the catheterism laboratory when performing ramp test during routine follow-up to optimize LVAD outflow or evaluate LV function recovery.

Some limitation of our work has to be acknowledged. Firstly, our framework was exclusively trained and run using numeric data. Obviously a next step will be to assess the performance of this framework using clinical data of pulmonary and systemic arterial pressure. Although this could be relatively challenging owing to the noise included in the signals under clinical acquisition, this limitation could be overcome by reducing the number of Fourier coefficients. Secondly, the 0D model used to train the DNN did not include possible alterations in left sided valve dysfunction such as mitral valve regurgitation, or right ventricle dysfunction, which may be both associated with LVAD implementation. Future refinements of our model should therefore take into account such possibilities.

## 5. Conclusion

In summary, we developed a novel method to assess systolic function of the mechanically assisted left ventricle, based on a DNN trained with data obtained from a 0D model of the cardiovascular system taking into account the presence of a LVAD. This DNN is fed with simple pressure signals (systemic and pulmonary) and with the rotation speed of the device, allowing to predict end-systolic elastance and other parameters of left ventricular function with excellent accuracy. Our method could represent a useful tool to optimize LV-LVAD interactions early after implantation as well as during chronic therapy, and to evaluate the possible functional recovery of the left ventricle.

## Data Availability Statement

The code used to support the findings of this study has been deposited in the following repository: https://github.com/matthiaszeller/VAD-0D-DNN.

## Author Contributions

JB: design and implementation of the work, data acquisition and interpretation, and manuscript drafting. MZ: implementation of the work, data acquisition and interpretation, and manuscript drafting. LP: data interpretation and manuscript drafting. SD: design of the work, data interpretation, final manuscript drafting, and financial support. LL: data interpretation, final manuscript drafting, and financial support. All authors critically reviewed and approved the final version of the manuscript.

## Funding

This work was supported in part by the Emma Muschamp Foundation, Lausanne, the Mahmoud Darvish Foundation, Lausanne, to JB, and from the Swiss National Science Foundation under project FNS-200021_197021 to SD.

## Conflict of Interest

The authors declare that the research was conducted in the absence of any commercial or financial relationships that could be construed as a potential conflict of interest.

## Publisher's Note

All claims expressed in this article are solely those of the authors and do not necessarily represent those of their affiliated organizations, or those of the publisher, the editors and the reviewers. Any product that may be evaluated in this article, or claim that may be made by its manufacturer, is not guaranteed or endorsed by the publisher.

## Footnotes

^1^ https://www.heartmate.com/healthcare-provider/resource-center
