# Supplementary material for: Deep Neural Network to Accurately Predict Left Ventricular Systolic Function Under Mechanical Assistance
Source: Front Cardiovasc Med. 2021 Oct 26;8:752088. doi: 10.3389/fcvm.2021.752088 (PMC8576185; doi:10.3389/fcvm.2021.752088)
Supplement: Supplementary file 1 [file Data_Sheet_1.PDF]

Supplementary Material

1 SUPPLEMENTARY TABLES AND FIGURES

1.1 Tables

	$E_{max,lv}$	$E_{max,lv,0}$	$G_{E_{max,lv}}$	$k_{E,lv}$
Lower bound	0.200	0.200	0.200	0.011
Upper bound	2.95	2.392	0.475	0.014

Table S1. Range of LV functional parameters in the 0D model used to model different degrees of heart failure (lower limit: severe heart failure, upper limit: normal systolic function). $E_{max,lv}$ [mmHg/ml], end-systolic left ventricular elastance; $E_{max,lv,0}$ [mmHg/ml], end-systolic left ventricular elastance in absence of baroregulation; $G_{E_{max,lv}}$ [mmHg/ml/(spikes/ml)], maximum baroreceptor gain; $k_{E,lv}$ [1/ml], steepness of end-diastolic pressure-volume curve.

#	layers	neurons	loss	loss_{val}	mae	mae_{val}
0	3	256	0.0077	0.0094	0.0560	0.0597
1	5	128	0.0091	0.0095	0.0605	0.0612
2	5	64	0.0102	0.0100	0.0642	0.0630
3	6	256	0.0082	0.0107	0.0575	0.0633
4	4	128	0.0087	0.0100	0.0593	0.0635
5	4	256	0.0084	0.0105	0.0587	0.0639
6	3	128	0.0085	0.0097	0.0587	0.0641
7	6	64	0.0104	0.0107	0.0647	0.0654
8	4	64	0.0112	0.0117	0.0671	0.0683
9	3	64	0.0107	0.0121	0.0669	0.0689
10	5	256	0.0079	0.0116	0.0566	0.0692
11	8	128	0.0101	0.0129	0.0636	0.0716
12	4	32	0.0133	0.0130	0.0736	0.0730
13	6	128	0.0095	0.0131	0.0623	0.0755
14	8	64	0.0122	0.0143	0.0698	0.0759
15	5	32	0.0153	0.0154	0.0786	0.0803

Table S2. Performance of different DNN architectures. Layers: number of hidden layers; neurons: number of neurons per hidden layer; loss: value calculated by the loss function; mae: mean absolute error. Subscript ”val” refers to the validation set. The Table is sorted by the validation mean absolute error. Only the best 16 architectures in terms of mae_{val} are shown.

1.2 Figures

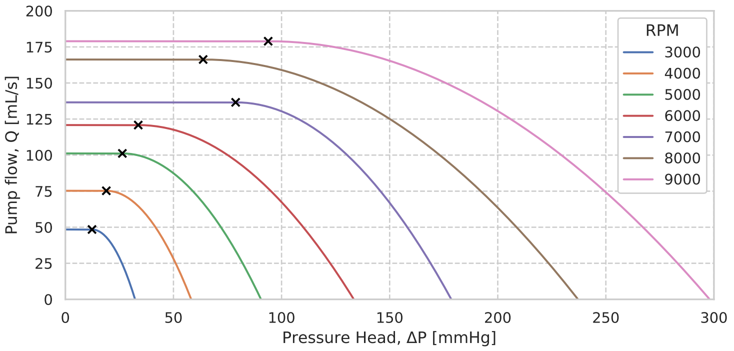

Figure S1. Pressure-flow curves of the HeartMate III LVAD. Flow rate (Q) is a function of the pressure difference (ΔP) across the pump. Black crosses indicate that the flow is constant below the corresponding ΔP. Abbreviation: RPM: revolutions per minute.

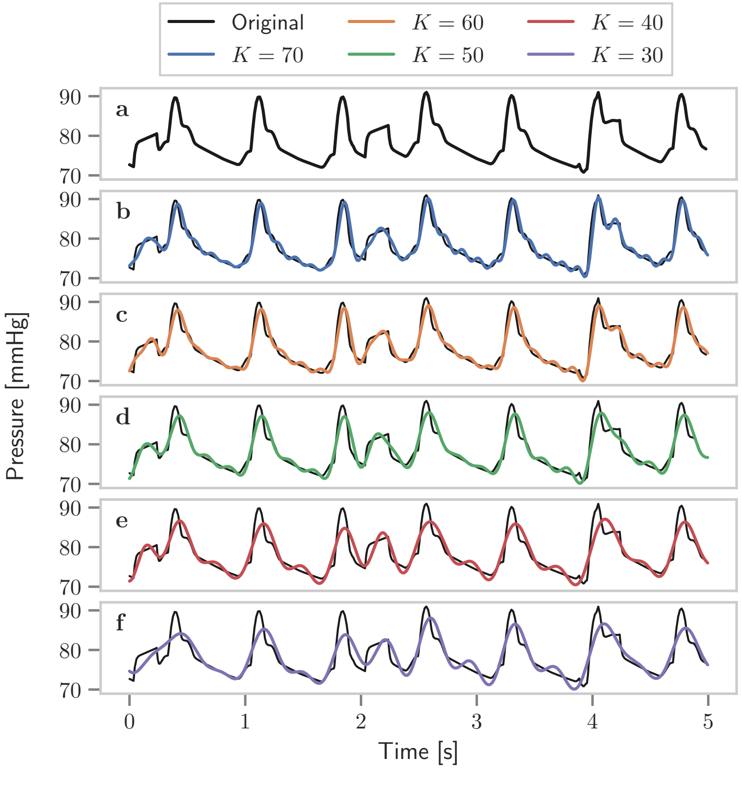

Figure S2. Reconstruction of the pressure signals in the time domain using different numbers of Fourier coefficients. Black curve **(a)** corresponds to a sample of the original signal of systemic arterial pressure (4000 RPM, severe heart failure). Curves **b**, **c**, **d**, **e**, **f** correspond to a reconstruction of the signal with 70, 60, 50, 40, and 30 Fourier coefficients, respectively.
