# Supplementary figures and images for: Deep Neural Network to Accurately Predict Left Ventricular Systolic Function Under Mechanical Assistance
Source: Front Cardiovasc Med. 2021 Oct 26;8:752088. doi: 10.3389/fcvm.2021.752088 (PMC8576185; doi:10.3389/fcvm.2021.752088)

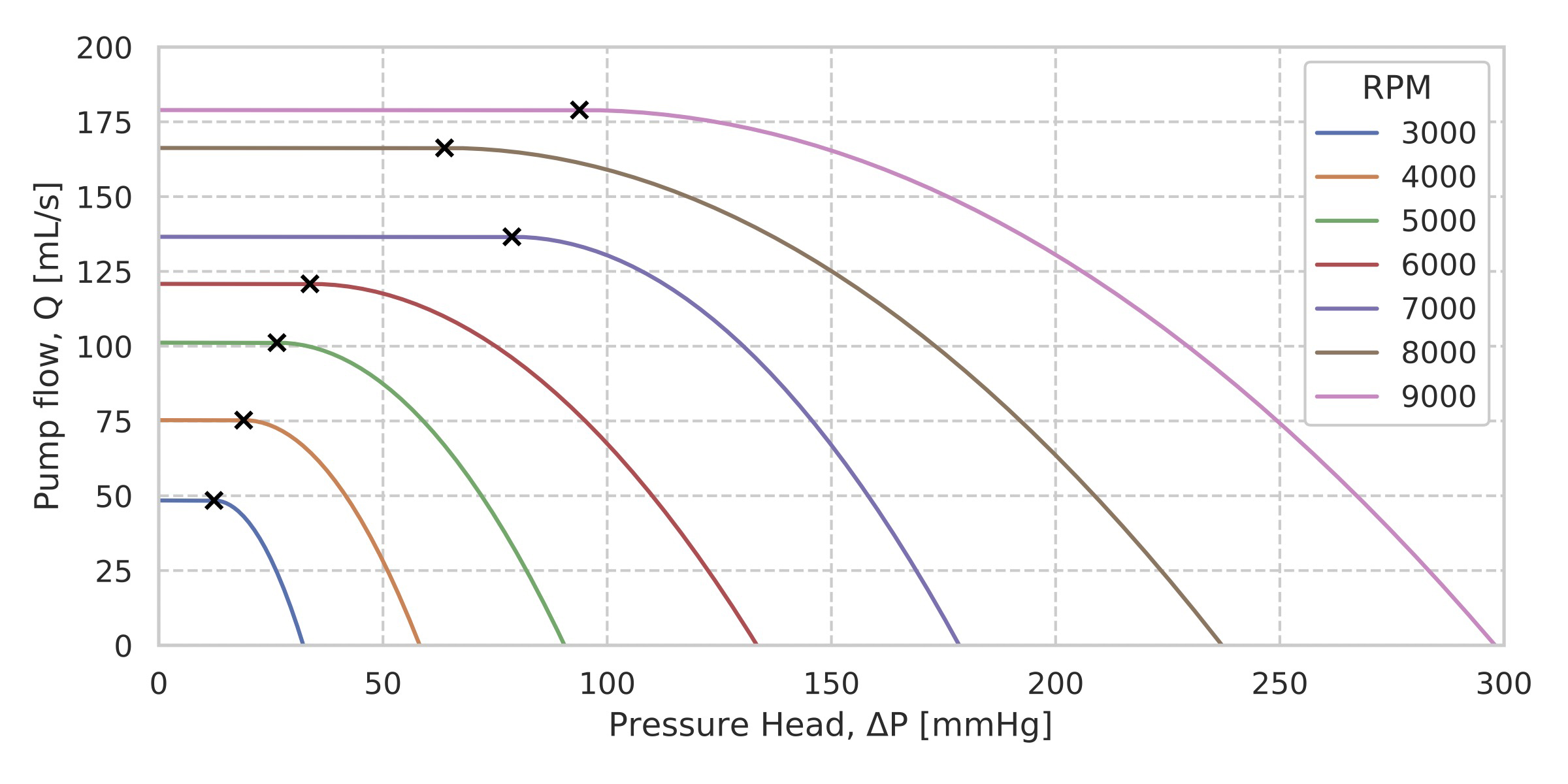

Supplement: Supplementary file 2 [file Image_1.JPEG]

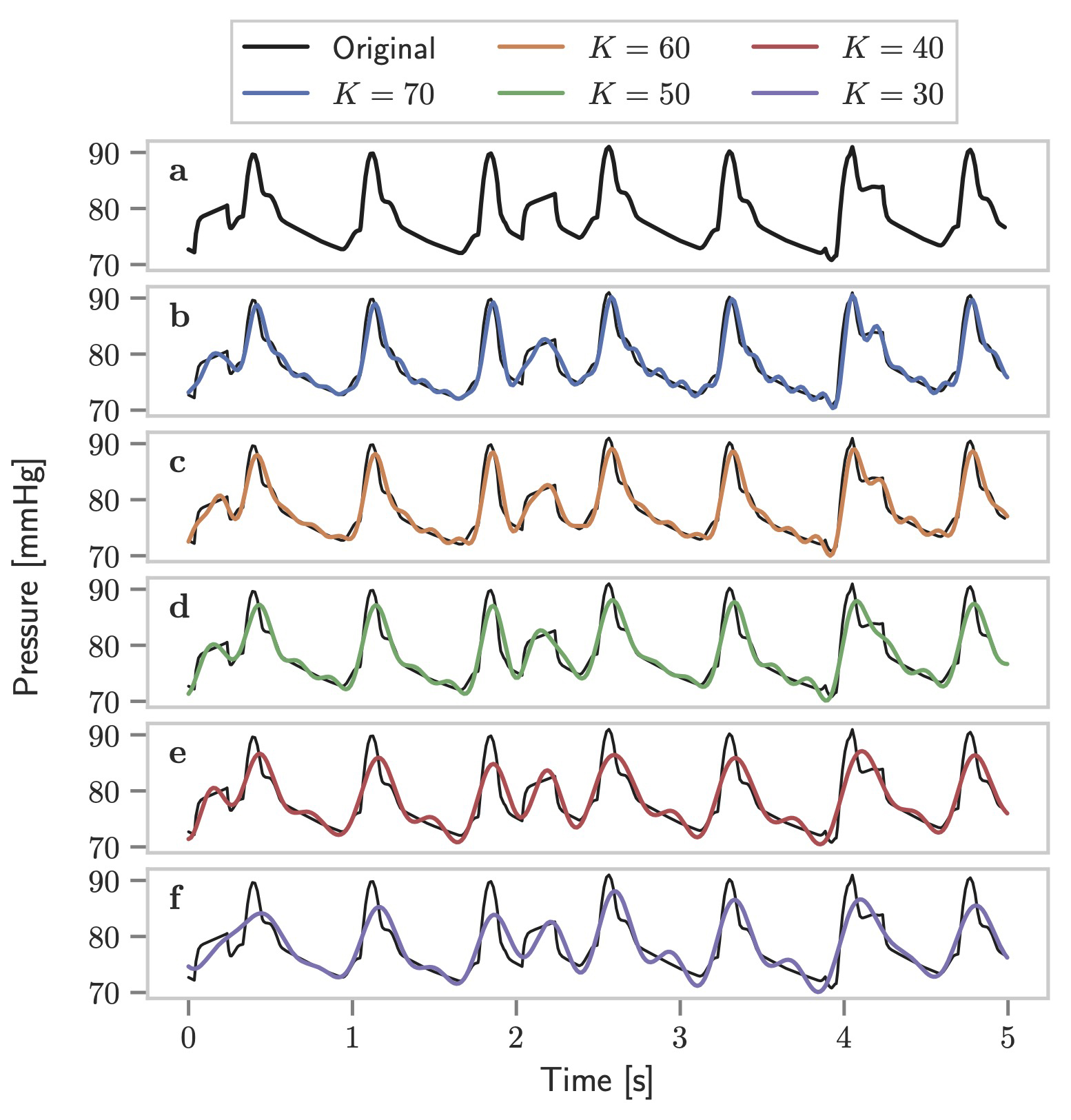

Supplement: Supplementary file 3 [file Image_2.JPEG]
